# Synthesis, characterization, and radiosynthesis of fluorine-18-AVT-011 as a Pgp chemoresistance imaging marker

**DOI:** 10.1038/s41598-022-22930-6

**Published:** 2022-11-03

**Authors:** Pardeep Kumar, Riptee Thakur, Pratap Chandra Acharya, Hosahalli K. Mohan, U. N. Pallavi, Divya Maheshwari, Afsal Mohammed K M, Aishwarya Kumar, Sridhar Goud Nerella, Raman Kumar Joshi, Manoj Kumar, Chandana Nagaraj

**Affiliations:** 1 Department of Neuroimaging and Interventional Radiology, NIMHANS, Bengaluru, Karnataka India; 2 Department of Pharmacy, Tripura University (A Central University), Suryamaninagar, Tripura (W) India; 3 Department of Nuclear Medicine, Sri Shankara Cancer Hospital & Research Centre, Bengaluru, Karnataka India; 4 Avaant Imaging, Lexington, MA USA; 5 Esente Healthcare, Bengaluru, Karnataka India

**Keywords:** Diagnosis, Predictive markers, Cancer imaging

## Abstract

P-glycoprotein (Pgp) is the most studied ATP-binding cassette (ABC) efflux transporter and contributes to chemoresistance. A few tracers have been developed to detect the in-vivo status of chemoresistance using positron emission tomography (PET) imaging. In our study, we have synthesized labeled AVT-011 with fluorine-18 (^18^F) followed by in-vitro and in-vivo analysis. Tosylate AVT-011 precursor was synthesized and characterized by ^1^H-NMR and ^13^C-NMR. AVT-011 was labeled with ^18^F using the nucleophilic substitution method, and a standard set of quality control was performed. The specificity for Pgp was tested in U87MG cells with and without an inhibitor (tariquidar). The biodistribution and in-vivo stability were tested in the small animals (mice). The biodistribution data of [^18^F]-AVT-011 was extracted from the PET-CT imaging of breast cancer patients (n = 6). The precursor was synthesized with 36 ± 4% yield and 97 ± 2% purity. The labeling was more than 95% with a 42 ± 2% yield, as evaluated by Radio-HPLC. The cell-binding assay showed a specificity of the tracer for Pgp as the uptake increased by twice after blocking the Pgp receptors. The radiotracer showed a hepatorenal excretion pathway for clearance in an animal study. The uptake was higher in the liver, lungs, spleen, and heart at 15 min and decreased at 60 min. The patients' distribution showed similar uptake patterns as observed in the small animals. [^18^F]AVT-011 was characterized successfully with high radiochemical purity and yield. The in-vitro and in-vivo studies proved its specificity for Pgp and safe for patient use.

## Introduction

Multidrug resistance (MDR) is the principal mechanism by which many cancers develop resistance to chemotherapy and is a significant factor in the failure of many forms of chemotherapy. Chemoresistance affects patients with various blood cancers and solid tumors, including breast, ovarian, lung, and lower gastrointestinal tract cancers. Tumors usually consist of mixed populations of malignant cells, some drug-sensitive while others are drug-resistant. Chemotherapy kills drug-sensitive cells but leaves behind a higher proportion of drug-resistant cells. Mainly, the cancers express MDR1 P-glycoprotein (Pgp) and its polymorphic variants^1–3^.

The major problem with tumors is either late diagnosis or chemoresistance to the therapy. No specific tool or diagnostic agent can assess chemoresistance at an early stage. A biopsy is considered a gold standard for detecting the tumor status using the molecular expression, but it has several limitations. The primary limitations involve accurate sampling or multiple sampling at various time points, which can cause stress to the patient. Nuclear medicine imaging is a non-invasive imaging technique that has the potential to image the molecular changes in the cell at an early stage. Few research groups have developed labeled anticancer drugs or molecules to detect cancer in precancerous and early cancerous stages^4,5^. ^18^F labeled erlotinib/gefitinib/doxorubicin has been developed as a radiotracer for evaluating in-vivo drug resistance^6–8^. Labeling the same drug used for chemotherapy makes more sense to predict its chemoresistance and may help differentiate between responders and non-responders.

ATP-binding cassette (ABC) family proteins, ABCB1 and ABCG2, contribute to multidrug resistance. In many studies, ABCB1/ABCG2 mRNA expression has been inversely correlated with the response to chemotherapy^9–11^. Therefore, the focus was shifted to image Pgp expression using PET imaging. Vlaming et al. (2015) synthesized [^18^F]-gefitinib and tested it in the cell lines overexpressing murine and human ABCB1 and Abcg2. The PET-CT imaging was done in the wild-type, Abcg2^−/−^, Abcb1a/1b^−/−^, and Abcb1a/1b; Abcg2^−/−^ mice. The results showed 2.3-fold increased brain levels of [^18^F]-gefitinib in Abcb1a/1b; Abcg2^−/−^ mice, compared to wild-type. Though the levels in the knockout animals were not different from the wild-type, showing that Abcb1a/1b and Abcg2 together limit access of [^18^F]-gefitinib to the brain.

[^11^C]-Verapamil (VPM) was developed as the first Pgp substrate radiotracer and is considered the most successful tracer for Pgp imaging. The applications of (R)-[^11^C]-verapamil in assessing the Pgp functions in different conditions like aging, depression, and neurodegenerative diseases have been reported and reviewed extensively^12^. Maria et al. (2020) have used [^11^C]-verapamil PET for evaluating Pgp function in drug resistance epileptogenic developmental lesions. They have conducted a dynamic [^11^C]-VPM-PET imaging on twelve healthy controls and two epilepsy patients. The imaging data were used to calculate the influx rate, VPM-K_1,_ using single-compartment modeling with a VPM plasma input function. Statistical parametric mapping (SPM) analysis showed a significantly lower uptake of VPM corresponding to the area of the epileptogenic developmental lesion compared to healthy controls^13^. Savolainen et al. (2020) used [^18^F]MC225 in mice and non-human primates, the dynamic PET images used for calculating the volume of distribution (V_T_), kinetic rate constants (K_1_, K_2_) in the baseline and post-inhibition scans. They found that K_1_ is the accurate parameter to measure the Pgp function. Still, it contradicted the other study carried out by different groups using [^11^C] metoclopramide that rely on K_2_ (efflux) as a measure of Pgp function^14^. Garcia-varela et al., 2021 have made a head-to-head comparison between (R)-[^11^C]-verapamil and [^18^F]MC225 in non-human primates for measuring P-glycoprotein function. In baseline scans, [^18^F]MC225 V_T_ values were higher and k_2_ values were lower than (R)-[^11^C]-verapamil, whereas k_1_ values were not significantly different. After blocking, V_T_ values were the same for both tracers, whereas the k_1_ and k_2_ values were higher for (R)-[^11^C]-verapamil than [^18^F]MC225. Their study concludes that k_1_ of (R)-[^11^C]verapamil may not be an adequate parameter to measure the Pgp function. The in-vitro studies showed [^18^F]MC225 to be more specific than (R)-[^11^C]verapamil^15^. Therefore, there is more to explore and understand the dynamics of the chemoresistance using Pgp as a marker. Kannan et al., 2020 have developed and characterized [^18^F]AVT-011 as a new radiotracer for imaging MDR in tumors. This study has shown the potential of [^18^F]AVT-011 to measure ABCB1 function in tumors. The study's major limitation was low yield, which may be due to multistep radiolabeling. Therefore, the authors emphasized developing a simple labeling method that can provide high yield and is feasible to translate into humans^16^.

We have designed, synthesized, characterized, and radiolabeled the tosylate precursor (AVT-011) using a single-step chemical reaction in the present work. The radiochemical purity, quality control, stability, animal bio-distribution, and breast cancer patients (n = 6) have been studied and reported in this paper.

## Material and methods

All the chemicals and reagents were procured from Sigma Aldrich (USA). ^18^F radioisotope was produced with our in-house 16.5 MeV Cyclotron (PETtrace 860, GE Healthcare, USA) by the proton bombardment on the enriched O-18 water using [^18^O(p,n)^18^F] reaction. The proton bombardment was done with the beam current ranging from 30 to 65 µA for 10–30 min depending upon the requirement of ^18^F. The ^18^F was delivered to the synthesizer module (Tracerlab FX2N, GE Healthcare, Chicago, USA) using Helium (UHP-6.0) as a carrier gas. All sep-pak cartridges like C18 plus, tC18 plus, Wax were procured from Waters-India, and C18ec was procured from Macherey–Nagel (Dueren, Germany). The pH was tested using pH paper (Fisher Scientific, New Hampshire, USA) and a pH meter (Mettler Toledo, Ohio, USA). The intermediate-I (4) was donated by Avaant Imaging (Lexington, MA, USA), and the precursor was synthesized by Essente healthcare (Bengaluru, Karnataka, India). The radiochemical purity was measured using high-performance liquid chromatography (HPLC) methods. HPLC system (Dionex, California, USA) was equipped with UV–Vis coupled and radioactivity detector. The quantitative analysis was done on C18 column (5 um 4.6 × 250, Shim-pack GW, Schimadzu) using mobile phase composition of acetonitrile (0.1% trifluoroacetic acid) and water (0.1% trifluoroacetic acid), starting with 5% acetonitrile (0–5 min), 5% to 100% acetonitrile (5–20 min), then 100% acetonitrile (20–25 min) and again at 5% acetonitrile (25-30 min). The residual solvents were measured using Gas chromatography (GC) (Scion 436 GC, Netherlands) with a flame ionization detector (FID). The column was operated initially at 40 °C for the first 3 min and then rose to 50 °C/min for up to 8 min, and the final temperature was set at 240 °C, and the column was BR-200 ms, 0.32 mm ID. The makeup gas consists of Helium (28 mL/min), zero air (300 mL/min), and hydrogen gas (30 mL/min) flow at the rate of 2 mL/min. The endotoxin test was carried out using Endosafe PTS cartridges on a NexGen Endosafe PTS machine from Charles River (Massachusetts, USA). A dilution factor of 100 was made for each preparation, 25 µl of the sample was added to each well, and a sterility test was performed using tryptic soya broth. PET-CT images were acquired on Philips Ingenuity TF 128 (Cleveland, OH, USA). It combines modular, LYSO-based PET components with a 128-channel CT component. The PET-CT imaging was done in breast cancer patients at Sri Shankara Cancer Hospital, Bengaluru, Karnataka, India. The biodistribution data of patients were used in our study.

### Synthesis of the ^18^F radiolabeled AVT-011 (7)

- ***Synthesis of 1,2-bis(tosyloxy)ethane (3)***

*p*-Toluene sulfonyl chloride **(1)** (570 mg, 3 mmol) was added portion wise to the mixture of ethylene glycol **(2)** (0.6 mL, 10 mmol) and pyridine (0.5 mL) at 0–5 °C and stirred for 3 h. TLC monitored the completion of the reaction. The reaction temperature was raised to room temperature and poured into ice water (50 mL) with constant stirring. A white solid was obtained, which was filtered and washed in cold water (50 mL). The solid was dried under air for 12 h to obtain the crude product. Then the crude product was purified by washing with ether to obtain 1,2-bis(tosyloxy)ethane **(3)** as a white solid (Yield = 72 ± 2%). HPLC and 1H-NMR characterized the product.

(b) ***Synthesis of toluene-4-sulfonicacid 2-{6-[2-(4-{4,5-dimethoxy-2-[(quinoline-2carbonyl)amino]benzoylamino}-phenyl)–ethyl]-3-methoxy5,6,7,8-tetrahydro-naphthalen-2-yloxy}-ethyl ester (5)***

**Figure 1:**
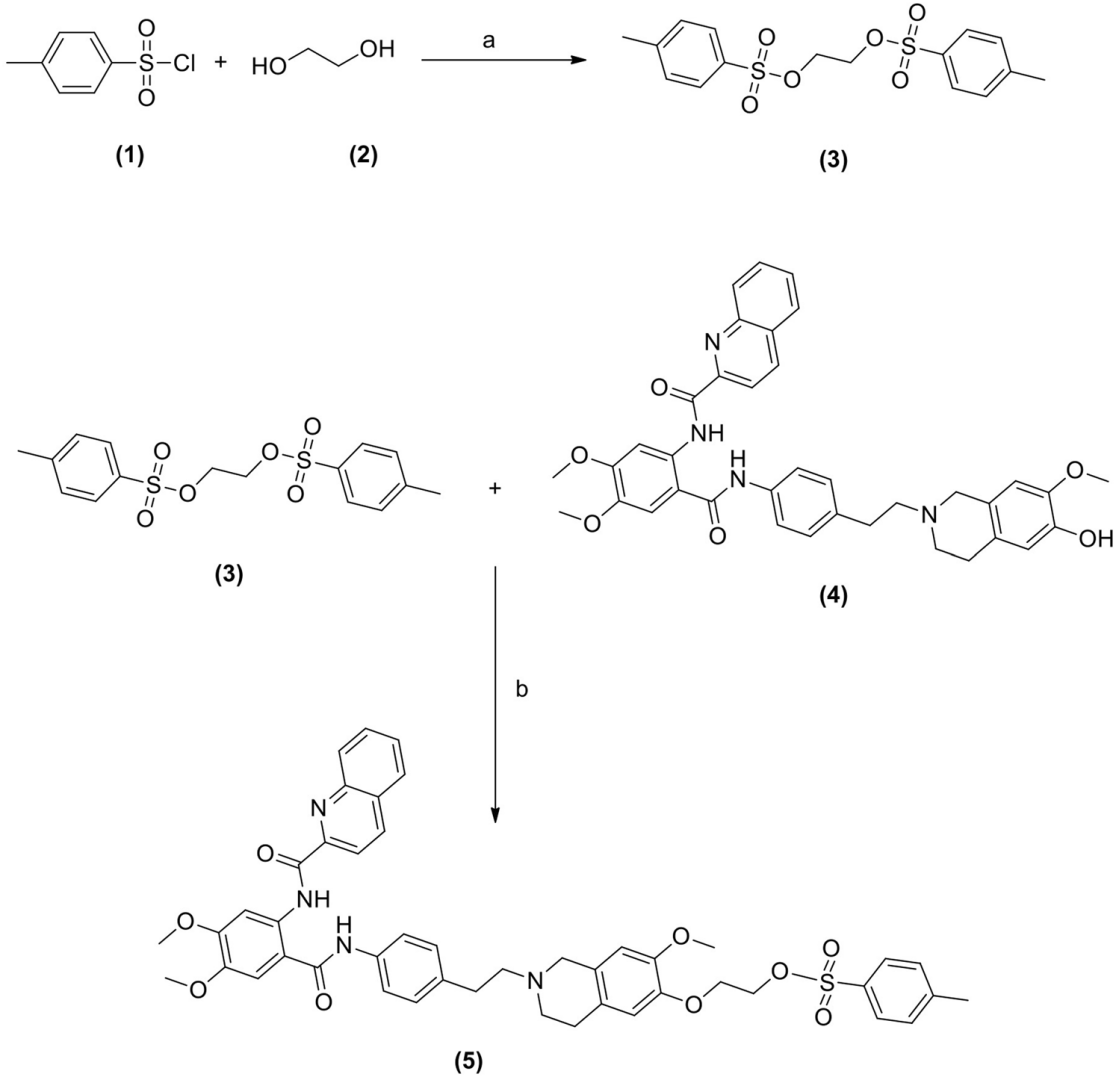
Reaction and reagents for the synthesis of the tosylate precursor **5**: (**a**) Pyridine, 0–5 °C, stirring, 3 h (**b**) acetone, Cs_2_CO_3,_ stirring at room temperature followed by heating and stirring at 50 °C for 6 h.

Purified intermediate, 6-O-desmethyl tariquidar (**4**) was donated by Avaant Imaging, MA, USA. 6-O-desmethyl tariquidar (**4**) (630 mg, 1 mmol) and 1,2-bis(tosyloxy)ethane **(3)** (440 mg, 1.2 mmol) were taken into a round bottom flask, and acetone (10 mL) and cesium carbonate (0.65 g, 2 mmol) were added at room temperature into the above mixture. The temperature of the reaction mixture was raised slowly from room temperature to 50 °C for 6 h. The complete scheme of reaction has been given in Fig. 1. The completion of the reaction was monitored by using a TLC. The reaction mixture was cooled to room temperature and filtered. The filtrate was concentrated to get crude product which was then purified by using column chromatography using 60–120 mesh silica gel as the stationary phase and 10% methanol in dichloromethane as the mobile phase to obtain the pure precursor AVT-011 (**5**). The precursor (5) was characterized by HPLC, LC–MS, ^1^H-NMR, and ^13^C-NMR.

(c) ***Fluorine-19 labeling and cold synthesis of AVT-011 (6)***

**Figure 2:**
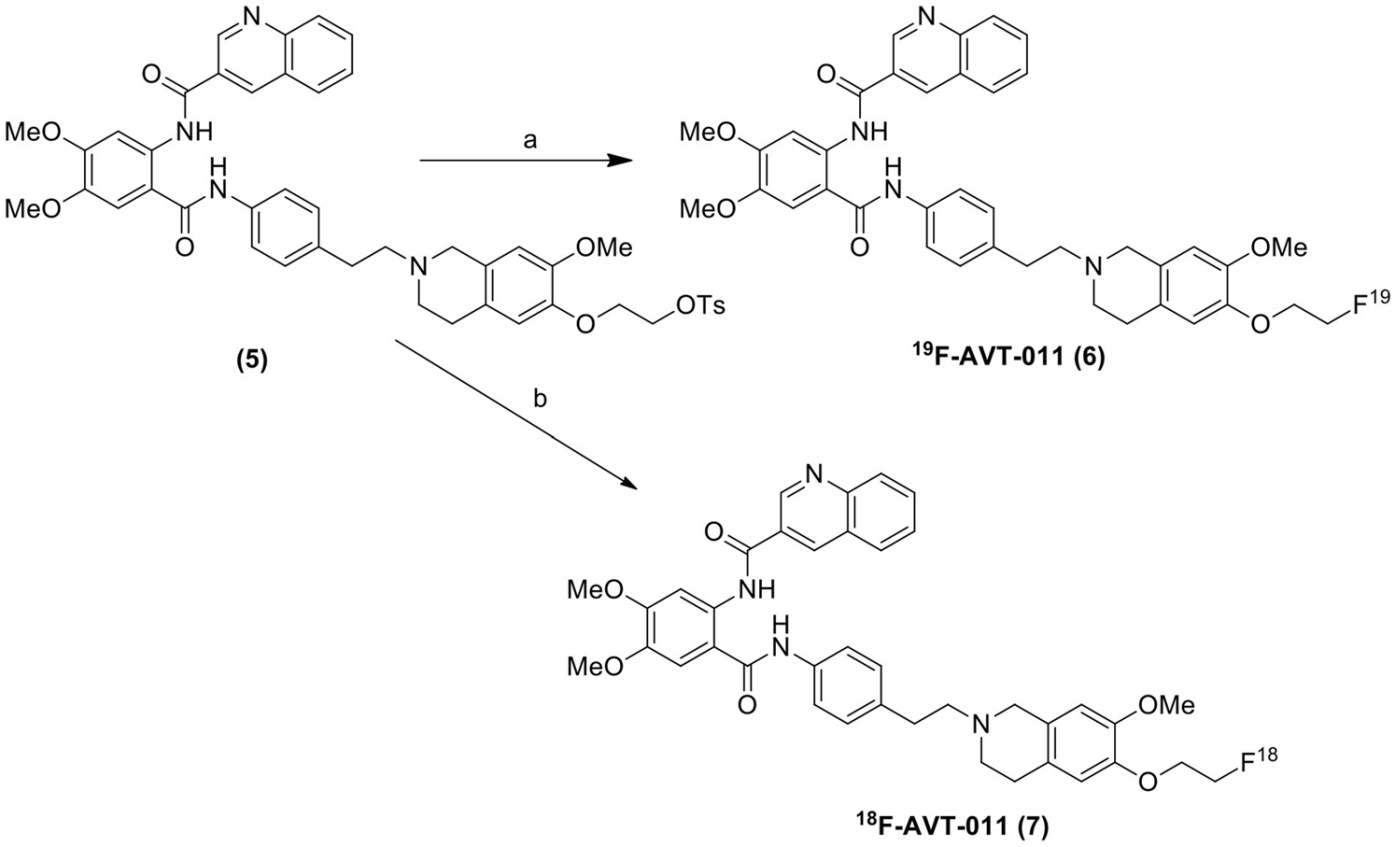
Reaction and reagents for the synthesis of the [^18^F]AVT-011 (**7**): (**a**) NaF, Kryptofix acetonitrile, 85 °C, N_2_ gas, 20 min. (**b**) ^18^F, Kryptofix K222, K_2_CO_3,_ acetonitrile, 110 °C, 10 min.

Tosylate precursor **(5)** (2 mg, 2.5 µmol) was mixed with sodium fluoride (NaF) (1.5 mg, 15 equiv.) and Kryptofix (3.6 mg, 4 equiv.) in the acetonitrile in a sealed vessel at 85 °C (in a pre-heated oil bath) for 20 min (Fig. 2). Afterwards, the acetonitrile was removed using nitrogen gas, and the residue was dissolved in ethyl acetate (1 mL). The suspension was loaded onto a silica gel cartridge (isosolute SPE column). The column was washed with ethyl acetate (4 mL) and followed by 10% ethanol in ethyl acetate (4 mL), and the final product was eluted with 40% ethanol in ethyl acetate (4 mL). The solvent was evaporated using nitrogen gas to yield the pure product (6**)** as a white solid (0.95 mg). The final product was characterised by LC–MS and ^1^H-NMR.

(d) ***Radiolabeling and synthesis of [***^***18***^***F]AVT-011 (7)***

The reactions were carried out in the FX2N tracer lab module; around 500 ± 50 mCi of^18^F was added to the chemistry module from the cyclotron. The ^18^F was trapped on a preconditioned anion exchange cartridge (QMA, ABX, Germany). It was eluted using a solution of kryptofix (7.5–15 mg of K222 and 1.5–3.0 mg of potassium carbonate) dissolved in 0.9 mL of acetonitrile and 100 µL of water. The precursor AVT-011 **(05)** (4–8 mg) was dissolved in 1.5 mL of solvent [dimethylformamide, dimethylsulfoxide, and acetonitrile (by heating at 80 °C)]. The reaction was carried out at 110, 130, and 160 °C for variable time points of 10, 20, and 30 min. The K222 eluent, precursor, and diluent (30% acetonitrile) were filled in vial-1, 3, and 5, respectively. Columns C18, tC18, and C18ec were used to purify the final product [^18^F]AVT-011* (07)*. The columns were preconditioned with ethanol (4 mL) and water (10 mL). The SPE cartridge was fixed between V17 and V15 at C18 (2) port. Water (4 mL), 8% ethanol (3 mL) and ethanol (1.5 mL) were filled in vial-12, 13 and 14 respectively.

### Quality control

The radiochemical purity of the preparation was estimated by HPLC, and the solvent phase was used as described in the materials section. The retention time of the cold AVT-011 standard was measured at λ_max_ 254 nm using a UV–Vis detector, whereas the [^18^F]AVT-011 **(07)** was detected using the radioactive detector. The quality control parameters like physical appearance, pH, radiochemical purity, radionuclide purity, chemical purities, residual solvents, sterility, endotoxin test, and stability *(in-vitro*) were done as per the standard protocol described elsewhere^17^.

### Cell binding assay

The cell binding was studied in the cancer cell line. U-87 MG cells were purchased from the National Centre for Cell Science (NCCS), Pune. The cells were cultured in Dulbecco's Modified Eagle Medium (DMEM)-high glucose containing 10% fetal bovine serum and 1% antibiotic in a T75 flask. The cells (suspended in media) were cultured in a T75 flask and placed in a humidified incubator at 37 °C under 5% CO_2_/95% air. The cells, when confluent, were detached using 0.25% trypsin–EDTA, washed and re-suspended in 1x  phosphate buffered saline (PBS) at a concentration of approximately 5 × 10^6^ cells/mL. Each test tube contained 1 mL of cell suspension into which 10 µL (~ 0.74 MBq) of [^18^F]AVT-011 was added and incubated for 15 min, 30 min, 60 min, and 120 min at 37 °C in a water bath.

The blocking studies were done by incubating the same cell suspension (1 mL) with 500-fold excess of Pgp blocking agent Tariquidar solution for 30 min at 37 °C in a water bath. After 30 min, 10 µL (~ 0.74 MBq) of [^18^F]AVT-011 was added and incubated for 15 min, 30 min, 60 min, and 120 min at 37 °C in a water bath. The incubation was terminated by adding 0.5 mL of normal cold saline. The mixture was centrifuged at 3000 rpm for 10 min, and the supernatant from each wash was collected into marked test tubes. The pellet was washed thrice with normal saline, and the supernatant was collected separately in the significant tubes. The radioactivity associated with cells and supernatant was counted in a gamma counter (Capnitec Inc), and the percentage of radioactivity bound to the cells was calculated.

### Animal studies

Animal biodistribution studies were carried out in normal Swiss albino mice (n = 12) after approval from the Institute animal ethic committee (NIMHANS no. AEC/70/453/NIIR dated 08.08.2019). All experiments were performed in accordance with the Committee for the Purpose of Control and Supervision of Experiments on Animals (CPCSEA) and complied with the ARRIVE guidelines. A group of mice (15–20 weeks, 25–35 g) were injected intravenously (in tail vein) with 4–6 MBq of [^18^F]AVT-011 diluted with saline (in a total volume of 100 µL), and the mice were sacrificed at 15-, 30-, 60- and 120-min post-injection. Mice were then euthanized by carbon dioxide inhalation, and tissues were dissected, washed free of blood, dried, and weighed. Concomitant radioactivity was counted with an ^18^F standard solution and injected dose per gram (% ID/g) was then calculated. For in-vivo stability, cardiac blood was collected (n = 2) in the heparinized vials after sacrificing the mice at 1 h. The blood was centrifuged at 3000×*g* for 10 min at room temperature to separate the plasma. An equal volume of separated plasma and chloroform/methanol (4:1, v/v) were mixed and centrifuged at 6000×*g* for 5 min to precipitate the proteins. The supernatant was subjected to the HPLC analysis.

### Human PET-CT imaging

The breast cancer patients were enrolled in the study before starting their chemotherapy after obtaining approval from the Institutional Ethics Committee (Sri Shankara cancer hospital and research center, Ref no. SSCHRC/IEC10/55 dated 20.08.2020). The patients were enrolled in the study only after signing the informed consent form (format enclosed), and all experiments were performed in accordance with IEC guidelines for human experiments. The vital parameters, including pulse, blood pressure, oxygen saturation levels, and respiratory rate, were measured in all the patients before and after injecting radiopharmaceuticals. Briefly, 370 ± 40 MBq of [^18^F]AVT-011 was injected intravenously in all the enrolled patients (n = 6). The whole-body images were acquired after 45–60 min post-injection. Firstly, a low dose CT was acquired without contrast followed by PET with a bed position for 3 min. The fused images were processed using iterative reconstruction in a dedicated 128 PET/CT ingenuity machine.

The images were analyzed for biodistribution of the tracer. The uptake in the various organs was measured and expressed in terms of standard uptake values (SUV). The background was measured over the lung fields where no lesion was identified. The SUV was measured over the organs like the liver, lungs, kidneys, intestine, brain, and lesion site. A standard 1 cm of the region of interest (ROI) was drawn on the ascending aorta to obtain SUV of the blood pool. The lymph node of the breast lesion side was considered for calculating SUV.

## Results

### Characterization of tosylate precursor and cold labeling

The compound 1,2-bis(tosyloxy)ethane **(**3) was synthesized with a yield of 72 ± 2%. The purity of the product was 99.3% as depicted by HPLC and ^1^H NMR (500 MHz, CDCl_3_) δ 2.46(s, 6H, –CH_3_), 4.18 (t, 4H, –CH_2_–CH_2_–), 7.34 (d, 2H, Aromatic), 7.73 (d, 2H, Aromatic). It was further conjugated with 6-O-desmethyl tariquidar (4) to form precursor (5) with a yield of 36 ± 4%. The purity of the precursor was 99.5%, as depicted by HPLC. The calculated ESI–MS (m/z) for C_47_H_47_N_3_O_9_S = 829.30 as compare to observe value of 829.35 [M]^+^, 831.40 [M + 2H]^+^. ^1^H NMR (500 MHz, DMSO-d_6_) δ 2.28 (t, 2H, –N–CH_2_–CH_2_–), 2.41 (m, 6H, –N–CH_2_–CH_2_–), 2.66 (s, 3H, –CH_3_–Ar), 2.82 (s, 2H, –N–CH_2_–), 3.69 (m, 4H, –O–CH_2_–CH_2_–), 3.88 (s, 9H, Ar–OCH_3_), 6.56–9.36 (m, 14H, Aromatic), 10.3 (s, 1H, –NH–). ^13^C NMR (125 MHz, DMSO-d_6_) δ 21.24, 21.58, 50.87, 56.11, 56.53, 66.94, 69.68, 105.66, 110.90, 112.48, 114.64, 122.08, 125.97, 127.00, 127.94, 128.10, 128.49, 129.28, 129.61, 129.79, 130.62, 132.08, 132.73, 134.31, 136.10, 136.15, 136.81, 138.01, 144.82, 144.92, 145.44, 145.62, 146.01, 146.29, 147.83, 149.10, 152.00, 163.50,167.46. The appearance of the singlet at δ 3.88 in the proton NMR confirmed the presence of the –OCH_3_ function. In contrast, the singlet at 2.66 ppm indicated the insertion of the tosylate bridge in precursor **5**. Aromatic protons were observed at δ 6.56–9.36, whereas the N-ethylene functions were found between 2.28–2.82 ppm in the ^1^H NMR. The appearance of the amide carbons at δ 163.50 and 167.46 ppm in the ^13^C NMR confirmed the formation of the precursor **5**.

The cold labeling was confirmed by a reaction between precursor (5) and sodium fluoride, which yielded ^19^F-labeled precursor [^19^F]-AVT-011 (06). The LCMS (ELSD) analysis confirmed that the isolated material was synthesized with high purity (99.4%). LCMS (ELSD): (m/z), calculated for C_39_H_39_FN_4_O_6_ = 678.29, Observed mass: 679.2 [M + H]^+^ , 700.2 [M + Na]^+^. ^1^H NMR (500 MHz, DMSO-d_6_) δ 2.44 (t, 2H, –N–CH_2_–CH_2_–), 2.78 (m, 6H, –N–CH_2_–CH_2_–), 3.64 (s, 2H, –N–CH_2_–), 2.92 (dd, 2H, –CH_2_–CH_2_–Ar), 3.78, 3.94, 4.04 (s, 3H, –O–CH_3_), 4.18 (t, 2H, –O–CH_2_–CH_2_–F), 4.35 (t, 2H, –O–CH_2_–CH_2_–F), 7.11–9.54 (m, 14 H, Aromatic), 1.25 & 12.50 (s, 1H, –NH–).

The feasibility of the precursor for labeling with ^18^F was evaluated by the reaction of precursor **5** with cold fluorine (^19^F) and is depicted in Fig. 2 (6). The LCMS data showed the substitution of the ^19^F with the tosylate group of the precursor. The appearance of a [M + H]^+^ peak at an m/z value of 679.25 in the mass spectrum confirmed the formation of the ^19^F labelled target derivative **6**. The yield of the target compound (**6)** was found to be 33 ± 4% and the product purity was greater than 99%. The chemical characterization data of the precursor and final compounds **3, 5, 6,** and **7** are given in supplementary Figs. 1–7.

### Radiochemistry

The various concentration of kryptofix and precursor were tried to obtain the molar ratio of AVT/K222_,_ which can provide the maximum yield. The data (Table 1) showed that the molar ratio of (0.25:1) AVT (8 mg)/K222 (15 mg) was appropriate to give the maximum possible yield. The suitable solvent for dissolving the precursor was acetonitrile (dissolved by heating), and the proper radiolabeling reaction temperature was 110 °C for 10 min (Fig. 2). After adding 500 ± 50 mCi of ^18^F, 210 ± 30 mCi of [^18^F]AVT-011 was obtained (decay corrected). The incorporation of the ^18^F into the precursor was 40 ± 4% as measured through HPLC. The final product was eluted with 1.5 mL of ethanol and further diluted with 15 mL of physiological saline and passed through a 0.22µ filter. After using various cartridges, C18ec was found to provide higher radiochemical purity with a yield of 42 ± 2% (Table 1). The molar activity was 700 ± 60 GBq/μmol. Therefore, the final loading conditions (in FX2N tracerlab module) were 15 mg of K_222_ (3 mg of K_2_CO_3_) in acetonitrile/water was loaded in tube 1. 8 mg of the precursor was dissolved in 1 mL of acetonitrile in tube 3. 5 mL of acidic water was loaded in tube 5, 5 mL of water, 3 mL of 8%ethanol and 1.5 mL of ethanol was loaded into the tube 12, 13 and 14 respectively. The preconditioned QMA was used for trapping ^18^F, and C18ec was used for final purification of the product from the reaction mixture. The product was collected in a collection vial containing 15 mL of saline and then transferred to the product vial by passing through a 0.22µ filter (cathivex).

**Table 1:** Showing different combinations to standardize the method yielding high radiochemical purity and yield.

	K222	AVT-011	Molar ratio AVT/K_222_	Solvent/reaction temp(ºC)/time(minute)/purification cartridge	% Yield/eluting solution	Radiochemical purity
1	7.5 mg (20 μmol)	8 mg (10 μmol)	0.5:1	DMF/160/30/C18 light	3 ± 2%, eluted with ethyl acetate	93 ± 3% (n = 3)
2	15 mg (40 μmol)	8 mg (10 μmol)	0.25:1	DMF/160/30/tC18 light	6 ± 3% eluted with 60% ethanol/water	95 ± 2% (n = 3)
3	15 mg (40 μmol)	8 mg (10 μmol)	0.25:1	DMF/DMSO/160/30/tC18 plus- 360 mg	7 ± 2%, eluted with 60% ethanol/water	97 ± 2% (n = 3) (n = 2, for DMSO)
3	15 mg (20 μmol)	8 mg (10 μmol)	0.25:1	ACN/110/10/C18ec-360 mg	42 ± 2%, eluted with ethanol (1.5 mL)	97 ± 3% (n = 8)

### Quality control

**Figure 3:**
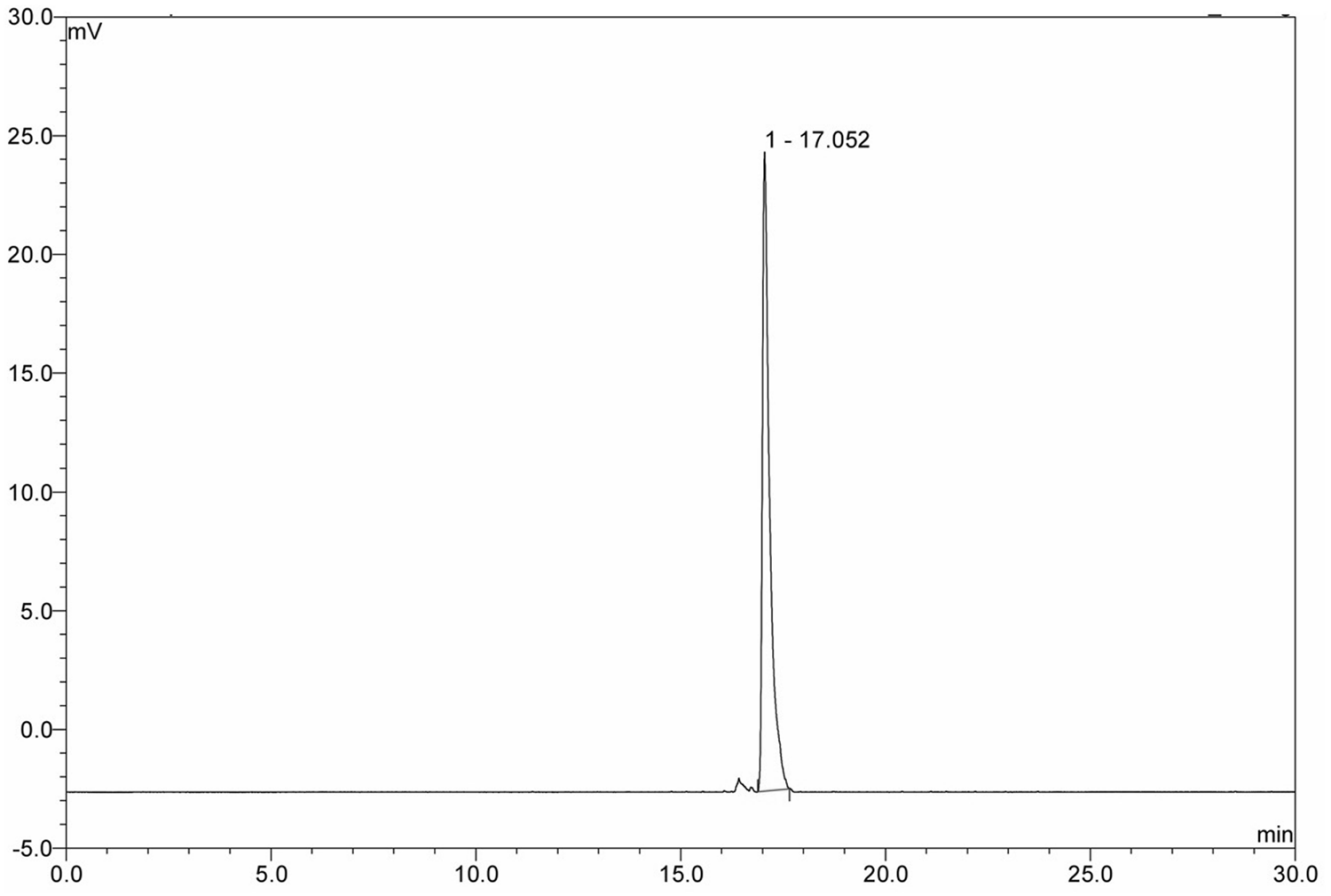
Figure showing radioactive peak for [^18^F]AVT-011 at 17 min.

**Figure 4:**
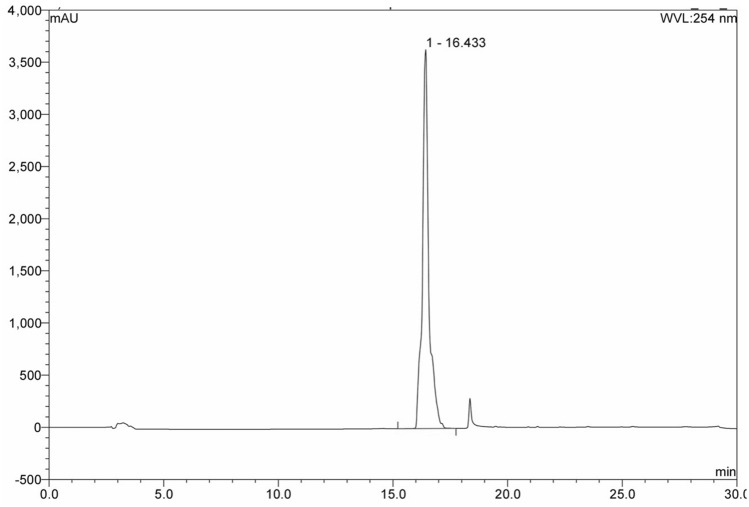
Figure showing UV/Vis peak for AVT-011 standard at 16.4 min.

The final solution appeared clear after physical inspection and had a pH of 5.5 ± 0.6. The radiochemical purity of the final preparation was 97 ± 2% as evaluated by HPLC with radioactive peak retention time at 16.4 ± 1.2 min (n = 8) as compared to free ^18^F at 4.0 ± 0.5 min (Fig. 3). The peak of [^18^F]AVT-011 was identified by comparing it with the UV/Vis peak of standard AVT-011 solution at 254 nm under identical conditions. The retention time of the standard AVT-011 solution was 15.8 ± 1.3 min (Fig. 4). The radiotracer was found to be stable under in-vitro conditions. The radionuclide purity was more than 95%, and a half-life of 111 ± 4 min proved the radionuclide identity as ^18^F with 511 keV peak as a significant peak. As evaluated by previously described methods, the kryptofix levels were below 50 µg/mL^17^. The concentration of residual solvent DMF was 80 ± 8 ppm (reference value-810 ppm). The ethanol content in the final preparation was 50,000 ± 10,000 ppm (reference value < 5000 ppm) due to the elution of the final product using ethanol. Still, the final preparation has less than 10% ethanol after dilution. The endotoxin levels were 1.0 endotoxin units (EU)/dose compared to the prescribed limit of 175 EU/dose. The preparation showed no turbidity in a soya broth over 14 days. These tests confirmed the sterility of the final dose and were suitable for patient use.

### Cell binding assay

The cell binding assay showed uptake of 41.2 ± 4.8% in the U87 cells at 15 min, decreasing to 30.4 ± 3.1% at 120 min (Table 2). The blocking study showed a significant (*p* < 0.005) increase in the radiotracer uptake by 40% with time. It showed that blocking the Pgp efflux pump leads to trapping the radiotracer inside the cells, and retention increases with time. It showed its specificity towards the Pgp.

**Table 2:** Comparison of cell uptake of [^18^F]AVT-011 between the U87 cells and blocked U87 cells (pre-incubated with Tariquidar solution).

Time (min)	U87 cells	Blocked U87 cells
15	41.2 ± 4.8	47.1 ± 1.3
30	36.3 ± 2.9	70.9 ± 4.3
60	29.4 ± 1.7	67.5 ± 8.1
120	30.4 ± 3.1	71.7 ± 8.1

### Animal biodistribution and stability

**Figure 5:**
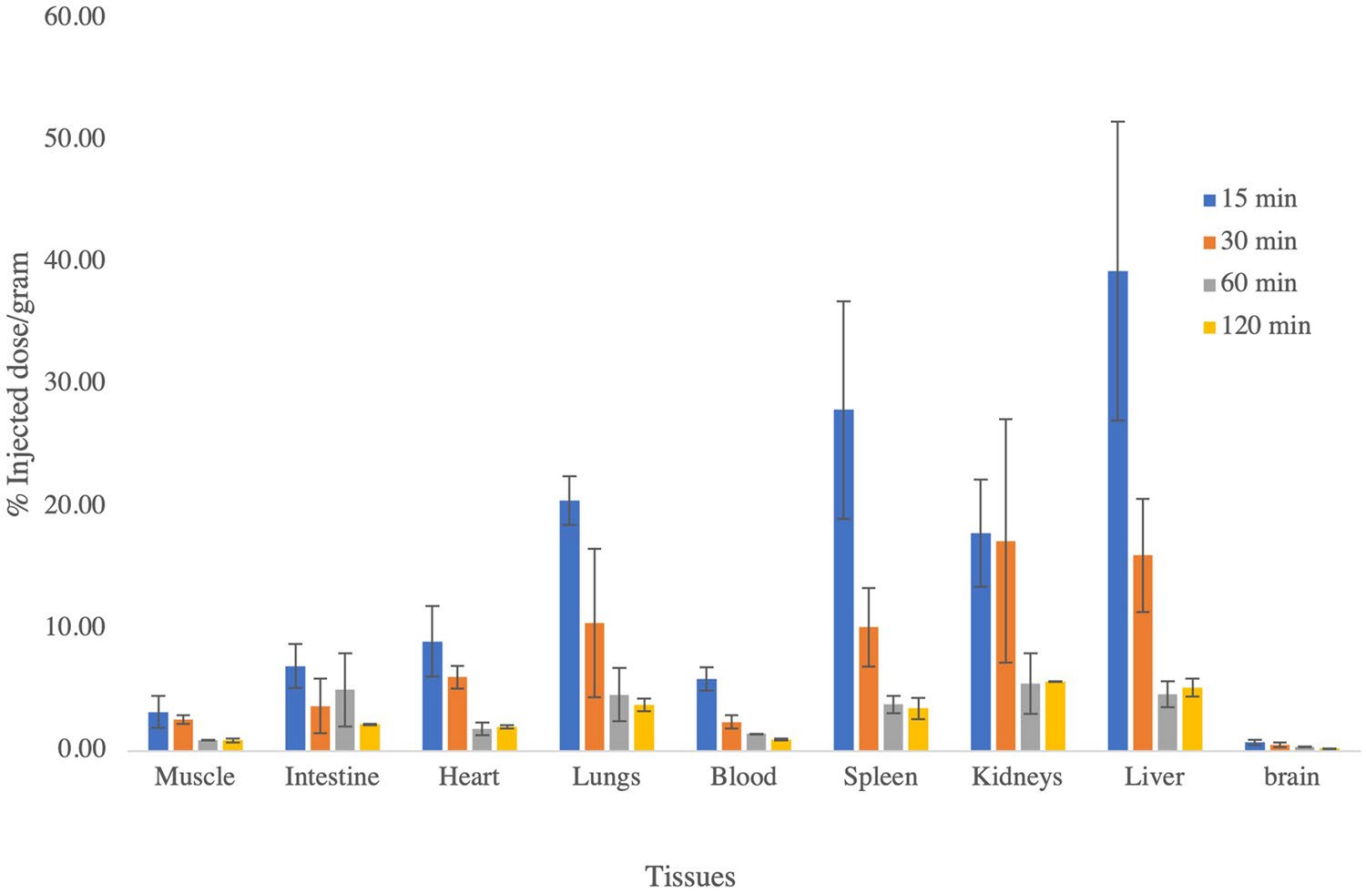
Diagram showing the distribution of [^18^F]AVT-011 in mice and pattern changes with time.

The tissue distribution showed the highest percentage of the injected dose was found in the liver (39.3 ± 12.2%), spleen (27.9 ± 8.9%), lungs (20.5 ± 1.9%), and kidneys (17.8 ± 4.4%) at 15 min (Fig. 5). With time, the activity was washed out from the liver, spleen, and lungs. The radioactivity increased in the kidneys and intestine with time. It showed a hepato-renal excretory route for the radiotracer. The normal brain showed minimal uptake of the radiotracer. The in-vivo stability was more than 92% at 1 h.

### PET-CT imaging

**Figure 6:**
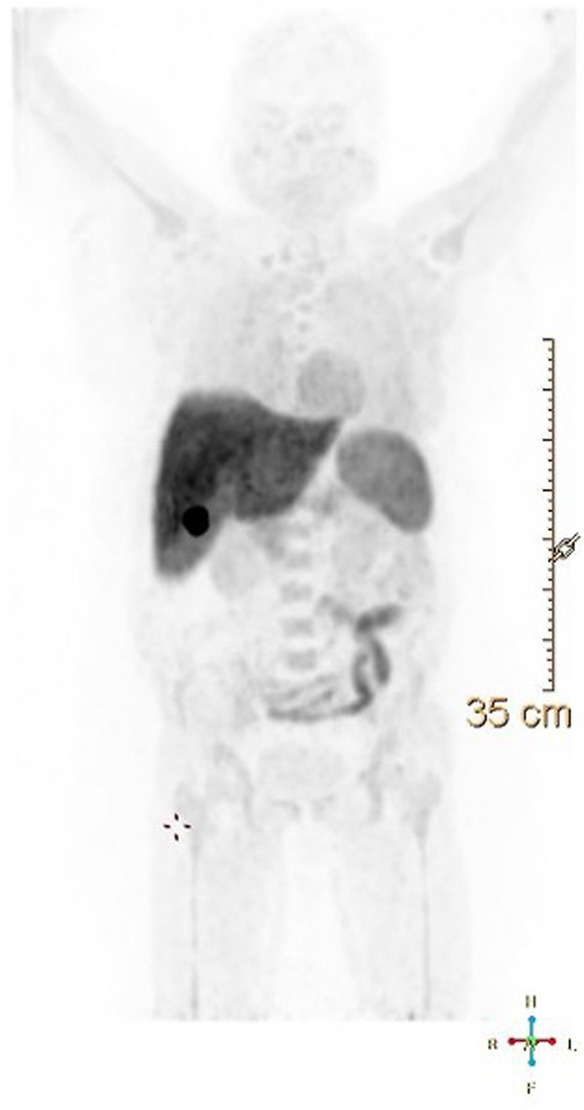
Figure showed the distribution of the [18F]AVT-011 in the patient post injection 60 min. The activity was maximally distributed in the liver, spleen, intestine, and kidneys.

There was a rapid clearance of the radiotracer from the blood pool with predominant entero-hepatic clearance and additional renal clearance, as noted in the animal studies. The highest concentration of the tracer was seen in the liver, spleen, colon, and myocardium. The SUV (Table 3) showed the highest liver and gall bladder uptake. The higher uptake in the liver may be due to the metabolic breakdown of the tracer in the liver; the activity was cleared from the liver through the gall bladder and intestine (Fig. 6). The uptake in the lesion site was low, but it must be correlated with the Pgp expression of the lesion from the biopsy sample to draw any conclusion.

**Table 3:** Showing SUV in the various organs.

Tissues	SUV
Breast	1.3 ± 0.1
Lymph node	1.6 ± 0.5
blood pool	1.5 ± 0.9
Bone marrow	3.8 ± 1.9
Gall bladder	78.3 ± 25.9
brain	0.2 ± 0.1
Liver	15.3 ± 4.2
Spleen	9.4 ± 4.1

## Discussion

Pgp plays a significant role in both physiological and pathophysiological conditions. It can efflux substrates out of the cell into the luminal space and ultimately out of the body. Due to the excretory role, it is majorly expressed on the cell membranes of the organs such as kidneys, liver, and intestine. The functional activity of Pgp is decisive under many conditions. The non-invasive imaging technique could provide insight into the functional status of Pgp behavior. Chemoresistance is a significant problem to the therapy, and timely detection of the chemoresistance may help the treating physician to change the course of the chemotherapy regimen. It can also help differentiate the responders from non-responders before chemotherapy starts. Therefore, many novel tracers have been developed and utilized in recent times. Kannan et al. have generated a new molecule, AVT-011, for imaging chemoresistance via targeting Pgp expression. Kannan et al. (2020) have used 6-O-desmethyl tariquidar as a precursor. First, they labeled ^18^F with 1,2-bis(tosyloxy)ethane to produce [^18^F]-fluoro (tosyloxy)ethane, which further reacted with a precursor to producing [^18^F]AVT-011. The study's primary limitation was a low yield of [^18^F]AVT-011. This may be due to the two steps involved in the labeling; despite low yield, they carried out animal studies. It showed higher brain uptake in the knockout (Abcb1a/b-/-) mice compared to the control mice. The blocking studies showed an eight-fold increase in brain uptake, proving its specificity. The doxorubicin-resistant mice showed 32% lower uptake and increased by 40% with tariquidar administration. Their results showed the specificity of the [^18^F]AVT-011 for the Pgp.

The main aim of our study was to overcome the limitations of the two-step method and develop a single-step synthesis method, and we have succeeded in our approach. We have conjugated the 1,2-bis(tosyloxy)ethane **(**3) with 6-O-desmethyl tariquidar to form a tosylate-based precursor (6). The tosylate group was substituted during the labeling by ^18^F, and a one-step radio-synthesis was developed. The precursor was characterized using HPLC, LC–MS, ^1^H-NMR, and ^13^C-NMR. It showed that the precursor was synthesized with more than 99% purity with a yield of 36%. Various kryptofix (7.5, 10, and 15 mg) were used to elute ^18^F from QMA cartridges, and maximum yield was obtained using 15 mg kryptofix and 3 mg of K_2_CO_3_. The molar ratio of 0.25/1 (AVT-011/K222) has given the maximum yield and radiochemical purity of 42 ± 2% with 97 ± 2%, respectively. Therefore, the final reaction condition was set to use 15 mg of kryptofix (3 mg of K_2_CO_3_) with 8 mg of precursor. [^18^F] was dried at 120 °C for 6 min, and the total elapsed time of the step was 20 min. The precursor was added to the dried [^18^F], and the reaction was carried out at 110 °C for 10 min. After completion of the reaction, the reaction mixture was cooled to 50 °C and diluted with 7 mL of acidic water. The diluent mixture was loaded onto the preconditioned C18ec cartridge and washed with 7 mL of water. The final product was eluted with 1.5 mL of ethanol in the collection vial and diluted with 15 mL of physiological saline. It is transferred to the sterile vial through a 0.22µ cathivex filter in the dispenser. The total time of the synthesis was around 40 min.

The in-vitro studies were carried out in the glioma cell line (U87 cells), which expressed Pgp receptors^18^. Therefore, we have used these cells for in-vitro validation. The cells were incubated with [^18^F]AVT-011 for a pre-set time, and uptake decreased with time and remained the same for 1 and 2 h, which proves its saturation at 1 h. It showed that the tracer was effluxed from the cell with time and maximum efflux was maximum at 1 h. Blocking the Pgp receptors by incubating with an excess of the tariquidar solution showed a increased uptake of [^18^F]AVT-011 in the cells. On blocking Pgp receptors, radiotracer gets trapped in the cells, which leads to an increase in the uptake. The uptake remained consistent at around 70% from 30–120 min. The in-vitro studies showed their affinity and specificity towards Pgp receptors. It proved the specificity of the [^18^F]AVT-011.

The animal biodistribution studies showed higher uptake in the liver, spleen, lungs, and kidneys at 15 min, even after 30 min, the uptake was higher in the liver, intestine, and kidney. This showed that the [^18^F]AVT-011 got metabolized in the liver and excreted through the intestine. The renal system was the other route of clearance. Hence, it showed the hepato-renal dual excretory pathway.

After obtaining institute ethics clearance, the tracer was tested on breast cancer patients (part of another research project). The biodistribution pattern in humans was found to be on similar lines with the animal distribution. The tracer was cleared through the hepatic-renal pathway. The uptake at the lesion site must be correlated to the Pgp status; hence it will be elaborated on in our other study. The human data showed the biodistribution of the [^18^F]AVT-011 in breast cancer patients and ensured human safety as all parameters were normal before and after the scan. The biodistribution pattern observed in our study was in similar lines as reported by Kannan et al. The major limitation of [^18^F]AVT-011 would be its uptake in the abdominal region. It cannot be an ideal tracer for imaging tumors located in the abdominal region, especially in the liver and gastrointestinal parts.

The affinity of [^18^F]AVT-011 for Pgp receptors has been proved in the previous studies conducted by Kannan et al., 2020. We have worked on the limitations pointed out in their studies and developed a single-step radiolabeling method with a high yield. We have increased the yield to 42 ± 2% as compared to 1–2% reported by Kannan et al. It has increased its chances to explore its clinical application. We are working further on using this tracer for gliomas and increasing its specific activity using the HPLC method of radiosynthesis. Though, solid phase purification is a convenient method and gives a high yield. Therefore, we have used this approach and developed a convenient, single-step method yielding high radiochemical purity.

## Conclusion

We have synthesized a modified precursor to developing a single-step radiosynthesis protocol for [^18^F]AVT-011 with a high yield and radiochemical purity. The high yield of the [^18^F]AVT-011 is advantageous for supplying this tracer at a distant hospital (without a cyclotron facility) and allows an exploration of its applications in various disorders.

## Supplementary Information

Supplementary Information.

## Data Availability

The chemistry workup was done at the chemistry lab, Esente healthcare, Bengaluru, and NMR data is available with them. All NMR data has been provided with the supplementary file. All radiochemistry and preclinical studies experiments were performed at the MR-PET Centre, NIMHANS, Bengaluru. Patient data is available with Sri Shankara cancer hospital, Bengaluru, Karnataka. The raw data is the hospital's property and cannot be uploaded. We have provided the image in the manuscript. Data are, however, available from the corresponding author upon reasonable request and with permission of MR-PET Centre, NIMHANS, Bengaluru, and Sri Shankara cancer hospital, Bengaluru, Karnataka.
