# Supplementary material for: Synthesis, characterization, and radiosynthesis of fluorine-18-AVT-011 as a Pgp chemoresistance imaging marker
Source: Sci Rep. 2022 Nov 3;12:18584. doi: 10.1038/s41598-022-22930-6 (PMC9633701; doi:10.1038/s41598-022-22930-6)
Supplement: Supplementary file 1 — Supplementary Information. [file 41598_2022_22930_MOESM1_ESM.docx]

**Suplementary figure 1: HPLC data of 1,2-bis(tosyloxy)ethane (3)**

**Suplementary figure 2: ^1^H NMR of 1,2-bis(tosyloxy)ethane (3)**

**Suplementary figure 3: LC-MS data of toluene-4-sulfonicacid 2-{6-[2-(4-{4,5-dimethoxy-2-[(quinoline-2carbonyl)amino]benzoylamino}-phenyl)–ethyl]-3-methoxy5,6,7,8-tetrahydro-naphthalen-2-yloxy}-ethyl ester (5).**

**Supplementary Figure 4:** ^1^H NMR of toluene-4-sulfonicacid 2-{6-[2-(4-{4,5-dimethoxy-2-[(quinoline-2carbonyl)amino]benzoylamino}-phenyl)–ethyl]-3-methoxy5,6,7,8-tetrahydro-naphthalen-2-yloxy}-ethyl ester **(5).**

**Supplementary Figure 5:** ^13^C NMR of toluene-4-sulfonicacid 2-{6-[2-(4-{4,5-dimethoxy-2-[(quinoline-2carbonyl)amino]benzoylamino}-phenyl)–ethyl]-3-methoxy5,6,7,8-tetrahydro-naphthalen-2-yloxy}-ethyl ester **(5).**

**Supplementary Figure 6:** ^1^H NMR of of ^19^F-AVT-011 **(6)**

**Supplementary Figure 7:** LC-MS spectra of ^19^F-AVT-011 **(6)**

**Supplementary figure 8: Cocktail HPLC of AVT-011 standard (17.1 min) and [^18^F]-AVT-011 (17.6 min)**

**
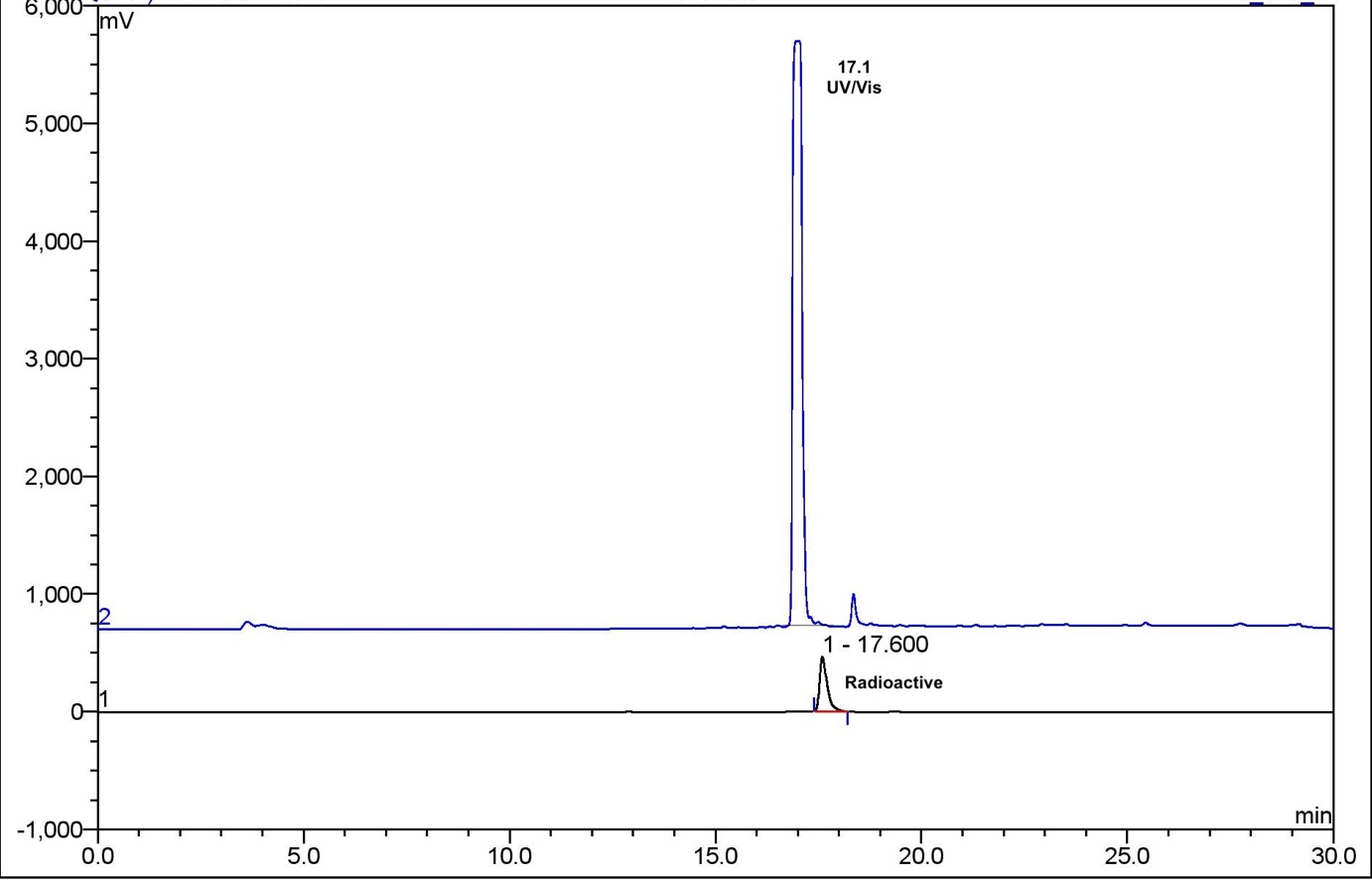
**
